# Digital twin assisted surgery, concept, opportunities, and challenges

**DOI:** 10.1038/s41746-024-01413-0

**Published:** 2025-01-15

**Authors:** Lisa Asciak, Justicia Kyeremeh, Xichun Luo, Asimina Kazakidi, Patricia Connolly, Frederic Picard, Kevin O’Neill, Sotirios A. Tsaftaris, Grant D. Stewart, Wenmiao Shu

**Affiliations:** 1 Department of Biomedical Engineering, Wolfson Centre, University of Strathclyde, Glasgow, UK; 2 Department of Surgery, University of Cambridge, Cambridge Biomedical Campus, Cambridge, UK; 3 CRUK Cambridge Centre, Cambridge Biomedical Campus, Cambridge, UK; 4 Centre for Precision Manufacturing, DMEM, University of Strathclyde, Glasgow, UK; 5 NHS Golden Jubilee University National Hospital, Clydebank, Glasgow, UK; 6 Department of Neurosurgery, Division of Surgery and Cancer, Imperial College Healthcare NHS Trust, London, UK; 7 Imaging, Data and Communications, The University of Edinburgh, EH9 3FG Edinburgh, UK

**Keywords:** Translational research, Biomedical engineering

## Abstract

Computer-assisted surgery is becoming essential in modern medicine to accurately plan, guide, and perform surgeries. Similarly, Digital Twin technology is expected to be instrumental in the future of surgery, owing to its capacity to virtually replicate patient-specific interventions whilst providing real-time updates to clinicians. This perspective introduces the term Digital Twin-Assisted Surgery and discusses its potential to improve surgical precision and outcome, along with key challenges for successful clinical translation.

## Introduction

The past few decades have seen radical advancements in both medicine and technology, drastically transforming the surgical field. The most prominent shift was the introduction of minimally invasive surgery (MIS), contributing to a significant reduction in trauma and complications for the patient, hence facilitating recovery periods, reducing hospital stays, and overall healthcare expenses^1^. Further advancements were realised by the incorporation of robotics and modern imaging technologies in MIS providing a greater degree of precision, better visualisation, and enhanced dexterity during surgery^2^. Nevertheless, such interventions comprise a series of limitations that have hindered their dominance in the surgical field, mostly associated with high costs and steep learning curves for practitioners to familiarise themselves with such techniques^3^. Moreover, surgical interventions, irrespective of the technology used, are not always successful, with the occurrence of surgical trauma remaining a major contributor to the mortality and morbidity of patients worldwide^4^. It is envisaged that surgical outcomes may be improved through the incorporation of innovative digital technologies. The use of such technologies is already gaining traction in the healthcare system, as this continues to shift towards a new era of digital transformation as a result of the recent pandemic. Therefore, implementing digital technologies in future surgery is inevitable^5^.

## The rise of Computer Assisted Surgery (CAS)

The use of digital or computer technologies to plan and perform high precision surgical procedures is now a well-established concept known as Computer-Assisted Surgery (CAS)^6^. This has been widely implemented in several surgical specialities, such as Computer-Assisted Implant Surgery (CAIS)^7^, orthopaedic surgery (CAOS)^8^, image guided surgery or neuronavigation in neurosurgery^9^, and so forth. CAS also known as ‘digital surgery’^10–12^ can be defined as ‘the use of technology for the enhancement of preoperative planning, surgical performance, therapeutic support, or training, to improve outcomes and reduce harm’^11^. This predicts the use of cutting-edge digital technology tools such as robotics, Artificial Intelligence (AI) and its subsets, and eXtended Reality (XR: virtual, augmented, mixed) to become major daily contributors in all phases of the surgical lifecycle.

Despite significant advancements of such technologies in surgical settings^13–18^, key challenges and limitations remain to be addressed prior to their widespread clinical use. For example, robotic-assisted surgery is well-known to have a lack of haptic feedback^19–21^, i.e., kinaesthetic (force) and cutaneous (tactile) feedback which are crucial sensory indicators for surgeons during surgery. The robot relies on visual and indirect/resistance feedback rather than true haptic feel. Integrating haptic sensors to robotic-assisted surgery has long been investigated^22–25^, and their incorporation in commercial surgical robots is still in its infancy. Recent research development in CAS has been mostly focussed on enhancing the level of assistance since current technologies provide very limited dynamic real-time intraoperative information^26^. Additionally, CAS technologies fall short in providing predictions to the surgical team^27^, and therefore, offer a limited contribution to on-the-spot decision-making. For example, when using XR technologies, there is currently a lack of interaction between the physical world i.e., what is happening in the operating theatre, and what is being shown virtually to the surgical team. Such challenges may be overcome by the emerging Digital Twin (DT) technology, that links together synergistically the physical and digital worlds, and is expected to be the new frontier in digital surgery.

## DT technology: background and healthcare applications

The history of DT technology dates back to 1970, when NASA employed on-ground simulators of the vehicle used for the Apollo 13 rescue mission. By rapidly adapting and modifying the simulations to closely match conditions as they were evolving in space, the first-ever DT was created. However, the term ‘Digital Twin’ was introduced much later in the 2000s by Prof. Michael Grieves^28^. Over the years DT technology has evolved greatly, and has been introduced in various industrial sectors, including product development^29^, manufacturing^30,31^, infrastructure^32^, and automotive industries^33^.

**Fig. 1:**
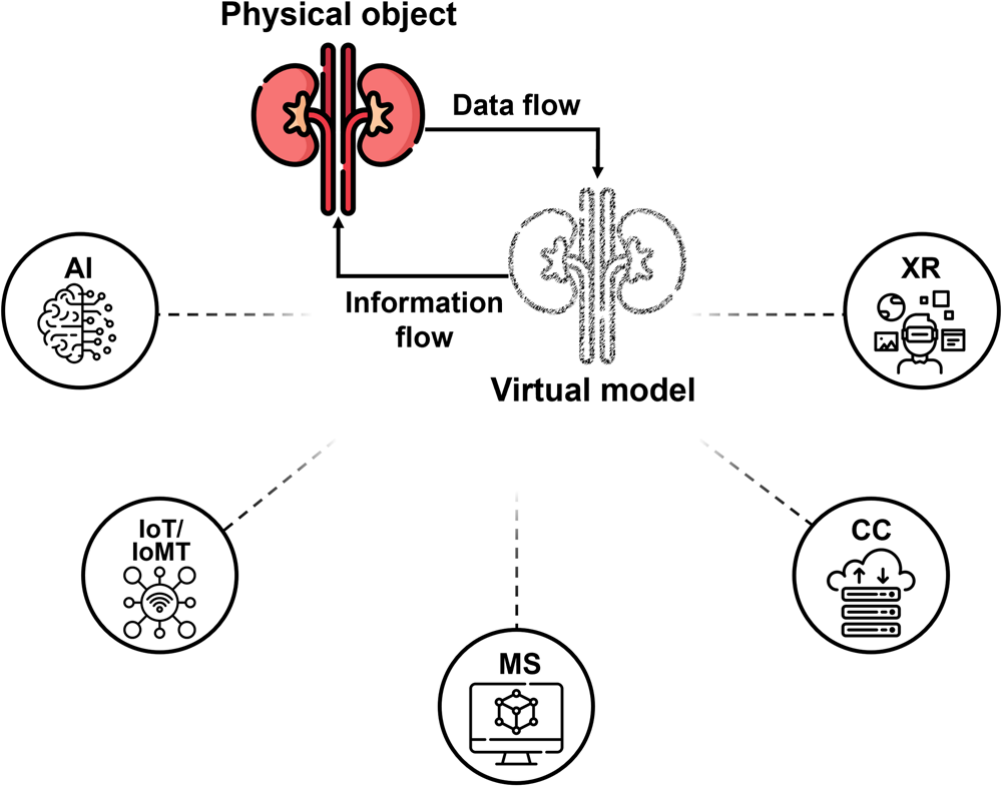
Schematic representation of the DT technology and its core technologies. A physical object is mirrored virtually with bidirectional real-time interactions (data and information flow). DT technology requires the following core technologies: Artificial Intelligence (AI), the Internet of (Medical) Things (IoT/IoMT), Modelling and Simulation (MS), Cloud Computing (CC), and eXtended Reality (XR). (Icons in figure were designed by Freepik and downloaded with permission from www.flaticon.com.).

DT technology involves a virtual model of real-world physical objects, processes, or systems based on collected data that is constantly updated and modified in real-time enabling their monitoring, evaluation, prediction, control and optimisation^34^. Therefore, any changes affecting the physical object are instantaneously mirrored in its virtual counterpart. A DT comprises three main elements: the physical object, the virtual model, and the technologies used to enable communication between the two^35^, as illustrated in Fig. 1. Modelling and Simulation (MS) technologies, including physical, mechanistic, and statistical simulations, play a leading role in DT construction since without these the virtual element of a DT cannot be realised^36,37^. AI in DT primarily involves the processing of the ‘big data’ associated with such a technology, which in turn provides insights, predictions, and suggestions, while XR-based technologies enable visualisation and user interaction with the virtual model. The Internet of Things, or in healthcare the Internet of Medical Things (IoT/IoMT)^38^, comprises network-connected devices, such as sensors, that are crucial for data collection, continuous data exchange, and communication between the twins. Computing power enables large volumes of data to be stored online, thereby widening accessibility even from off-site locations. These are all essential components for data collection and storage, which ultimately provide real-time performance and feedback through interactions between the physical and virtual models^34^.

DT technology holds great promise in virtually mirroring parts of the human body, from individual cell function to complex tissues and organs. Yet the primary and more ambitious objective is to virtually replicate an entire human body that is patient-specific, thus creating a Digital Human Twin (DHT)^39,40^. DT technology offers numerous opportunities in healthcare^41^, for example in medical devices to gain insight into their in vivo functionality and therefore predict future outcomes for the patient, and hospital facilities to manage resources^42^ and ensure smooth workflow in wards and clinics^43^.

## Digital Twin-Assisted Surgery (DTAS)

Herein, we propose the term “Digital Twin-Assisted Surgery (DTAS)” that integrates DT technology in CAS for assisting perioperative processes to enhance surgical training, planning, precision, safety, and patient care. The novelty of DTAS lies in the real-time virtual model showcasing the interplay between the “objects”, in this case the patient/patient organs being operated on (e.g., kidney, brain, eye) and their physiological parameters (e.g., blood pressure, heart rate, oxygen levels), and the “processes” i.e., surgical intervention (e.g., cutting, suturing, ablation).

**Fig. 2:**
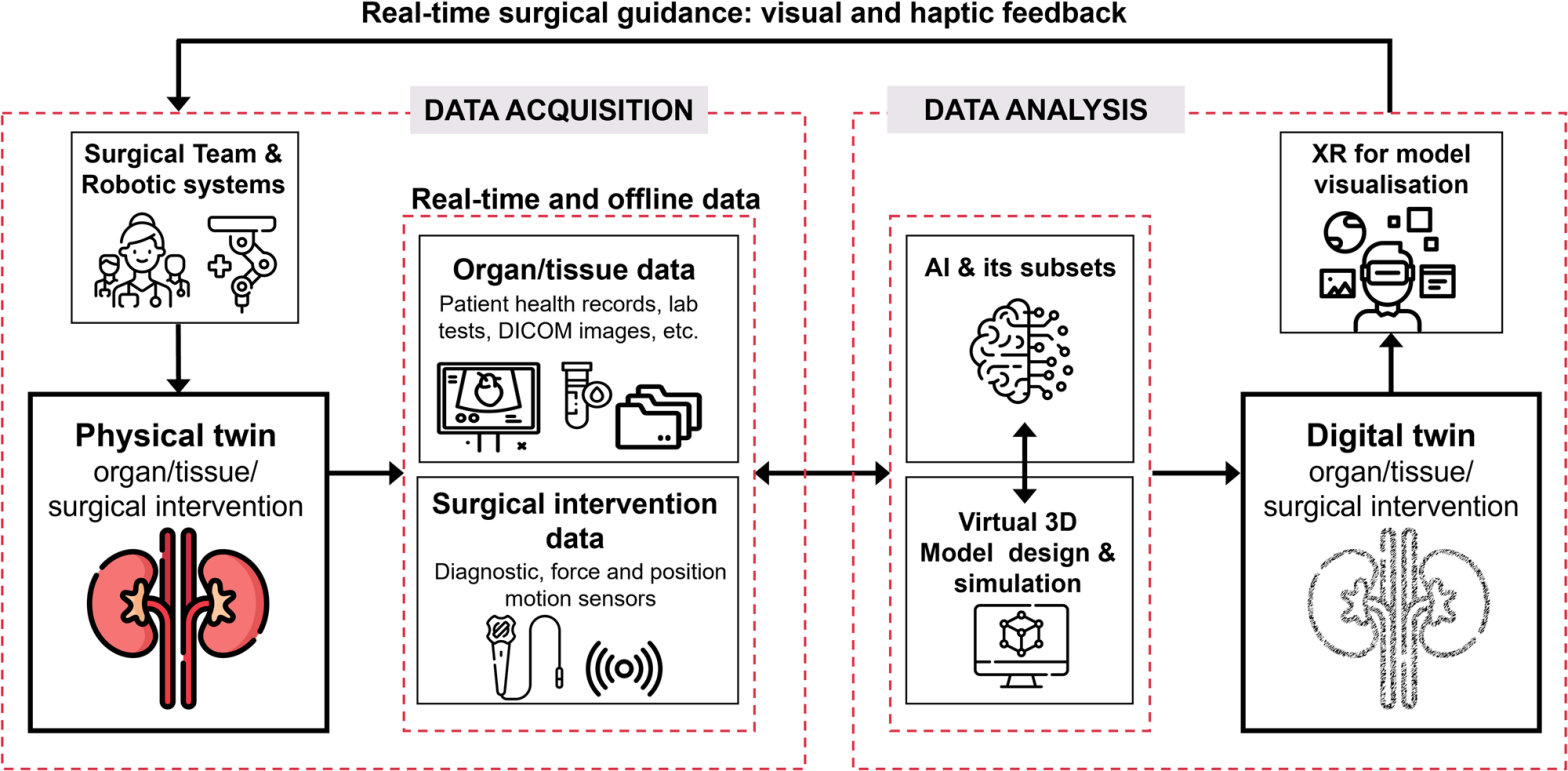
Digital Twin-Assisted Surgery (DTAS) proposed concept. Data is obtained from the physical twin both in real-time and prior to the generation of the digital twin (DT) (offline). This data is analysed using artificial intelligence (AI) and implemented to develop models and simulations, ultimately contributing to the generation of the DT. The DT provides real-time surgical guidance, such as visual and haptic feedback to the surgical team or surgical robot. Extended Reality (XR) may also be used by the surgical team for better visualisation and interaction with the DT. (Icons in figure were designed by Freepik and downloaded with permission from www.flaticon.com.).

DTAS comprises similar elements to other DT applications, as schematically illustrated in Fig. 2. Data is an essential element of a DT irrespective of the target application; therefore, the first stage of DTAS is data acquisition from the physical twin (i.e., tissue, organ, surgical instruments, or intervention) that may be obtained prior to the generation of the virtual DT, or when this is offline, and whilst the DT is active i.e., in real-time. Generally, such data is acquired from medical images (DICOM files from CT, MRI, X-ray, ultrasound), electronic sensors (wearable, physiological, optical, mechanical, and positional), medical devices (glucose and blood pressure monitors), patient health records, molecular and genetic biomarkers, and lab results (blood tests)^44–46^. This may be compiled and stored in electronic databases e.g., using cloud-based storage systems, rendering it accessible to the team responsible for 3D model representation and construction, clinicians, and hospital personnel.

The data is analysed via AI and used for the construction of the virtual model^46^. For example, Deep Learning (DL) methods may be implemented to automatically segment clinical CT or MRI scans, facilitating the reconstruction of the DT 3D geometry^47,48^. Thorough data analysis is crucial to ensure that: (1) there are no datasets that could introduce bias to the system, (2) all data records are complete and therefore do not promote skewness, (3) there are no inconsistencies that could lead to a weak DT model, and (4) any missing important data fields that could hinder the model’s validity are identified and incorporated. Additional data to run reliable simulations within the clinical timeframes may be based on information from the literature e.g., in vivo and ex vivo studies involving human tissue mechanical characterisation and blood flow dynamics measurements such as cardiac output, while biomechanical, mathematical, and statistical modelling are implemented to emulate physiological conditions. For high-fidelity computational simulations, including finite element analysis (FEA), computational fluid dynamics (CFD), fluid-structure interaction (FSI), fast reduced order models (ROM) calculations, the biomechanical and physical system properties and associated boundary conditions and modelling parameters must also consider factors such as age, gender, heart rate, and organ/tissue pathology which highly influence physiological behaviour^45^.

Once the virtual model is established, XR technologies may be used by the surgical team for better visualisation and interaction with the model with the additional capability of providing haptic feedback. With real-time data acquisition and analysis, the surgical team is provided with live updates, thus facilitating intraoperative decision-making^49^. In real-time DTAS, the DT takes the role of a navigator that maps out different surgical routes and possible outcomes to the surgical team—analogous to the Maps applications in our mobile phones where different routes are mapped out to the desired destination that instantaneously update according to one’s location. For instance, in this case, the DT would provide information and predictions on tissue cutting routes to avoid inadvertent damage, and blood flow alterations and blood loss, e.g., when a vessel is clamped or an organ is partially severed^50,51^. Preoperatively, DTAS allows the surgical team to virtually attempt new techniques or explore different access points and routes prior to the actual intervention on the patient. Postoperatively, DT could facilitate the generation of patient- and case- specific documentation which can contribute to the development of a virtual surgery database^45^. Given the predictive nature of such technology, DT may also be used for long-term treatment of patients post-surgery^52^. This may involve abnormality predictions and early disease detection allowing for timely changes in medication and surgical reintervention plans. Ultimately, DTAS shall contribute to enhanced surgical accuracy, and therefore, minimal complications, and a reduction in recovery time for the patient.

### Applications of DTAS in various surgical specialities

The concept of DT technology to inform surgical procedures has already been proposed in various specialities^53^. Table 1 presents some of the recent work published in this area, however given the infancy of DT in surgery, most investigations involve early components of a DT, for example, material constitutive models or specific algorithms towards the generation of a functional DT.

**Table 1:** An overview of recent studies proposing or implementing DT technology in surgery

Study ID	Year	Study type	DT, technology, and application	Impact of DT on current surgical practice
**Orthopaedics**				
Aubert et al. ^90^	2021	Concept	DT: Tibial fractures Technology: patient-specific FEA biomechanical modelling (stress distribution, fracture stability and bone healing process). Application: to evaluate the biomechanical properties of different stabilisation methods on bone healing.	Providing quantitative information to clinicians thereby assisting their decision-making during orthopaedic surgical trauma procedures.
Ahmadian et al. ^91^	2022	Feasibility	DT: vertebroplasty procedure Technology: Deep convolutional Generative Adversarial network and finite element modelling. Application: to analyse the vertebra’s behaviour to variations in mechanical loading, predict its fracture response, and link this to vertebroplasty parameters (e.g., needle tip location, cement volume, and injection rate).	Help clinicians assess the effect of vertebroplasty procedures on the mechanical integrity of the vertebra in cancer patients presenting with lytic metastatic tumours.
**Oncology**				
Shi et al. ^92^	2022	Concept	DT: the abdomen, including the liver, blood vessels, and tumours. Technology: optical marker tracking, correlation methods, and holographic augmented reality. Application: to include the effect of respiratory movement, and the internal heterogeneous structure of the abdomen during percutaneous puncture in liver tumour surgery.	To provide the surgical team with better visualisation of minimally invasive procedures involving percutaneous needle punctures (e.g., thermal ablation) by providing accurate intraoperative information on tumour position as well as blood vessel distribution to enhance procedure safety.
**Cardiovascular surgery**				
Rouhollahi et al. ^48^	2023	Concept	DT: aortic valves. Technology: a fully automatic deep learning AI tool, coined CardioVision (CV). Application: to analyse calcification distribution on patients with severe aortic stenosis.	For the clinicians and surgical team to better understand aortic valve disease, and therefore assist with predicting adverse events, selecting the most appropriate type of surgery as well as being able to carry out timely and informed decisions.
**Neurosurgery**				
Shu et al. ^49^	2023	Concept	DT: coined ‘Twin-S’ mirroring skull-based surgery Technology: high-precision optical tracking markers and real-time simulation. Application: to provide a virtual representation of the surgical tool, patient anatomy, and surgical camera during skull-based surgeries.	To assist surgeons with complex brain surgery particularly image-guided interventions, e.g., mastoidectomy. This framework provides real-time surgical information and virtual model updates.
**Minimally Invasive Robotic Surgery (MIRS)**				
Hagmann et al. ^58^	2021	Feasibility	DT: telesurgery robotic system (DLR MiroSurge) Technology: real-time monitoring of the robot (robot-centric DT), asynchronous multi-body framework physics simulation to track present objects and their poses (object-centric DT). Application: to monitor robotic surgical training and provide haptic assistance.	To help surgical trainees practice basic skills, such as pick, place, and path following, when using a robotic system. Ultimately, this DT technology is predicted to support surgeons during MIRS by providing individualised and contextual assistance to enhance surgical performance.
Bonne et al. ^70^	2022	Feasibility	DT: robotic surgery basic surgical tasks. Technology: novel framework designed for performing surgical peg transfer. Application: to assess the impact of network variations i.e., instability issues, on telesurgery performance.	To assist remote robotic telesurgery and overcome latent network challenges.
Cai et al. ^59^	2023	Feasibility	DT: robotic surgery coined ‘Virtual reality-based DT robotic minimally invasive surgery (VRDT-RMIS)’. Technology: design of the robot’s remote centre of motion algorithm and hardware-software framework for real-time control of DT and haptic feedback. Application: to simulate surgical skills training including peg transfer and soft tissue cutting tasks.	To provide a surgical training simulating platform equipped with high-refresh-rate haptic feedback that is currently lacking in most RMIS simulators.

Apart from research investigations, review papers have been published proposing DT frameworks for specific organs, such as the brain^54^, and surgical procedures^55–57^, highlighting the benefits of DT not just as a surgical assistant, but also as a predictive tool for pharmacological interventions, and for better understanding of organ function (healthy) and dysfunction (pathologies). Moreover, DT’s potential for providing both haptic and visual feedback has also shown great promise in mirroring procedures where this is lacking, such as MIS and robotic surgery^58^, and is anticipated to facilitate the training of such interventions by providing surgeons with sufficient motor skills to control a surgical robot^58,59^.

For instance, in orthopaedics DiGioia and Jaramaz^60^ introduced the concept of “closing the loop” which meant that CAS would not only contribute to intraoperative adjustments but also postoperative monitoring. DT and DTAS will further offer continuous feedback through real-time data from implantable sensors and biometric devices on recovery and complications. Data that will inform postoperative care, adjustment of rehabilitation plans, and refinement of future interventions linking surgical planning, execution and outcome. “Closing the loop” will enable real-time data-driven adjustments, allowing for more personalised treatment strategies and the better care of patients.

Moreover, DTAS also holds immense promise for improving outcomes in complex oncological surgeries and organ-sparing techniques. For example, this technology can be explored in colorectal surgery, hepatopancreatobiliary (HPB) surgery, and urological surgeries such as kidney and bladder cancer surgeries. In colorectal surgeries, the DT can integrate imaging data from CT and MRI to create a virtual model of the colon and surrounding tissues, mapping the exact location of tumours and critical structures like blood vessels^61^. Surgeons can then use this to plan the optimal resection strategy, minimising damage to healthy tissue. This same approach can be utilised in HPB surgery. Here, surgeons can simulate different resection strategies to maximise tumour removal while preserving liver function. In addition, DTAS can predict how much functional liver tissue will remain after resection, helping avoid post-hepatectomy liver failure. This can also be implemented in kidney-sparing surgeries, such as partial nephrectomies by creating a detailed 3D model of the kidney, tumour, and vasculature. Surgeons can simulate the best cutting plane, ensuring they remove the tumour with clear margins while preserving as much of the healthy kidney as possible. Real-time updates of imaging data^62,63^ can help to guide ablation procedures such as radiofrequency or cryoablation by showing precise tumour boundaries and thermal damage areas, preventing injury to nearby organs while also ensuring complete ablation of cancerous tissue. Incorporating DTAS into surgery could significantly improve patient outcomes, reduce surgery times, minimise risks, and allow for more personalised and evolving treatment approaches^61^ with the implementation of new ergonomic paradigms enhancing surgical performance^64^.

### Potential in surgical education and training

DT technology is poised to revolutionise surgical teaching and training, particularly through the integration of advanced robotic-assisted devices that enable multiple operators to collaborate effectively in confined surgical fields^18^. Traditionally, surgical training has been based on a ‘see one, do one, teach one’ approach coupled with countless repetitions^65^. Considerable changes have already been implemented through the introduction of AI and XR technologies^66,67^. However, DT has the potential to take the simulation technologies a step further. By constructing a surgical DT, a repository of surgical knowledge may be generated (e.g., through data from previous surgical performances) that could contribute to the standardisation of certain surgical procedures whilst also making this information accessible to the wider surgical community including experts from across the globe^5,55^.

DT in surgery allows clinicians to virtually practice complex surgical procedures in a safe, controlled, and realistic environment^53,55^. This could benefit surgeons irrespective of their level of expertise. DT also provides a platform for surgeons to experiment on surgical cases that are essentially impossible to work on in real-life situations, for example in pregnancy and foetal surgery.

In the same way that current image guided surgery informs and guides the surgeon anatomically, DT will be able to provide additional feedback to the surgeon but will have the benefit of real-time updated information so as to make surgery more precise rather than less precise as the surgery progresses and the anatomy changes. This will make high risk procedures much safer and provide the blueprint or ‘missing link’ for more complex robotic surgery.

In light of this, the introduction of such modern technologies calls for radical changes in the way the surgical training curriculum is devised. As highlighted by the Royal College of Surgeons (England) Future of Surgery report^5^, surgical training must strive to keep on evolving with the advances in modern technology and take on a multi-disciplinary approach by incorporating knowledge of computing, engineering, and data literacy. However, this does not stop with novice surgical trainees, experienced surgeons must also keep up with new, emerging technologies and surgical techniques.

### Potential in telesurgery

The rapid advancements in network communication, such as the latest 5 G and 6 G communication technologies, have been fundamental towards the development of functional DTs, given how much this technology relies on rapid, real-time communication. The use of 5 G communication has also made significant improvements in the field of telesurgery, primarily in minimising latency^68,69^. Despite this, the translation of telesurgery on humans still faces significant challenges, mostly associated with communication networks such as latency and instability, especially in remote, underdeveloped countries^70,71^. The implementation of a DT framework comprising AI-based intelligent systems could address such limitations owing to its ability to automatically update based on the virtual model simulations and physical sensor observations, rather than completely relying on the clinicians at the surgical site and those assisting remotely, therefore requiring less data interchange over the network^69,70,72^.

## Challenges to clinical translation

While DT has the potential to offer significant benefits and improvements in surgery owing to its non-invasive, controllable, and repeatable nature^73^, there still exist key challenges that must be overcome prior to successful clinical translation.

### Technical challenges of DTAS

#### Data related challenges

Issues concerning the translation of DT technology in the medical field, including surgery, are predominantly data related. Contrary to the industrial sector, where DTs mirror non-living objects such as machines, DTs in surgery would mirror complex surgical interventions based on human data, from physiological parameters to complex organ and tissue conditions, to surgical robots and instruments. Such data is collected from a wide range of sources, and in most cases, this is often sparse, noisy, or captured in an unstructured manner, for example, health records that are not digitised, thus adding to the complexity of data acquisition, storage, and analysis^40,74^. Therefore, reverse-engineering would have to be employed to obtain the appropriate data for the construction of an accurate and reliable DT model^75^. One way to facilitate this is by creating data-sharing platforms between institutions^76^, for example from clinics and hospitals, to collect and exchange patient health records, anonymising them when necessary. There is also a need for verification, validation, and uncertainty quantification of the DT model prior to implementing it for clinical decisions and predictions^40,74,75^, in addition to frequent model recalibrations to match the physical twin based on age, geographical location, gender, and race, to ensure precision and an unbiased treatment^37,74^.

#### Advanced sensor technology challenges

Achieving a fully-functional surgical DT, also calls for the development of advanced sensor technologies, including sensors to monitor the surgical process (e.g., tool-tissue interactions)^77^ which would be useful in providing haptic feedback, and wearable sensors to obtain specific patient health information^78^. For example, this would involve research on the development of novel materials and the incorporation of microelectromechanical systems (MEMS) for the fabrication of soft, flexible sensors^25,79^, research in nanotechnology to develop fast response miniature sensors, and advances in microfluidic-based sensor technologies^80^.

#### Infrastructure and resources challenges

In a real-time DT, considering that data is continuously fed into the system as the DT model actively updates, a large amount of data is generated that needs to be effectively analysed and stored, requiring state-of-the-art computational power and infrastructure that supports such ‘big data’^81^. Moreover, to construct and maintain a surgical DT, the input of experts from diverse backgrounds, including surgeons and clinicians, engineers, and data analysts, is required. Thus, this entire process can be time-consuming, and energy- and resource- intensive, which might prove costly and challenging when scaling-up to widespread use in healthcare systems, especially in the case of a real-time DT where computational and network speed are crucial^74^. Therefore, the right support from companies investing in this technology and the computational framework behind it is required, as well as support from governments, regulatory bodies, and insurance companies to exploit the full benefits of a surgical DT. Ultimately, the goal of DTAS is twofold, to: (1) improve patients’ outcome, and (2) enhance surgical precision through personalised planning (assist the surgical team). Therefore, it is essential to take this into account when developing surgical DTs and ensure that they are implemented where they would drastically contribute to this goal.

### Ethical considerations

Data-driven approaches such as surgical DTs, raise a lot of ethical questions^82^, even more so with DHTs^39,83^. This is mostly due to the acquisition of copious amounts of personal and sensitive data, that could threaten the patient’s privacy if not handled securely and in an ethical manner. Thus, it is imperative that patients are always provided with informed consent and full transparency, i.e., why is the data being collected, and how will it be managed and shared^12^, even if the DT will mirror parts of the human body, e.g., organ DTs. This is especially important when real-time data is acquired and fed to the DT during surgery while the patient is unconscious. Following consensual data acquisition, robust cybersecurity measures should be in place to prevent data breaches from cloud-based storage systems, and the misuse of data^84^. In addition to this, in order to enforce privacy and increase patient trust, sensitive patient data must also be encrypted and equipped with access restrictions^40^. From the patient’s perspective, the introduction of DTs might evoke the fear that the surgeons are being replaced by machines and computerised models^37^. Thus, all parties involved in the development of DTs must understand that this technology should by no means replace the surgeon and their expertise but rather assist the surgical team in providing better care for the patient, hence the introduction of the term DTAS. In medicine, the importance of doctor-patient confidentiality is well-established, and this must also be considered in the development of surgical DTs.

### Regulatory and legal challenges

Legal frameworks are already in place regarding the collection of patient health data. In the UK this is achieved according to the Data Protection Act of 2018, which implies that there should always be a valid lawful basis as to why data is collected, ultimately to safeguard patient’s privacy. However, similar to the EU’s General Data Protection Regulation (GDPR) and the US’ Health Insurance Portability and Accountability (HIPAA), questions have been raised whether this is enough to keep up with the data-rich, digital transformation^85^.

Moreover, given that a surgical DT is essentially a medical device, regulatory compliance is key. However, given the infancy of such a technology in the medical field, currently there are limited regulations in place that enforce legal liability with the implementation of DT in surgeries, or rather DT in healthcare as a whole^12^. The novelty of digital technologies in this sector spurs several uncertainties for regulatory bodies, as well as the need for substantial resources to evaluate the associated risks, and thus, regulatory clearances are typically granted depending on the application. Some of the few existing regulation clearances include the approval granted in September 2022 for AI-driven clinical trials to Unlearn.AI by the European Medicines Association (EMA) (www.unlearn.ai), and the recent clearance by the US Food and Drug Administration (FDA) in March 2024 to inHEART for their AI-driven software to create 3D cardiac models (www.inheartmedical.com). Despite these advancements, there is still a long way to go, therefore it is crucial for major regulatory bodies (the EMA, FDA, and the Medicines and Healthcare products Regulatory Agency (MHRA)) to be involved in DT projects to witness first-hand the DT development, which in turn could facilitate the regulatory pathway. This is already being exploited in major DT projects such as the Living Heart project by Dassault Systèmes where the FDA has been a major collaborator since 2014 (www.3ds.com/heart).

Thus, moving forward, as the DT technology becomes increasingly integrated within the healthcare system, and indeed the whole surgical lifecycle, it is envisaged that ethical and legal issues concerning the use of DTs in clinical settings are properly addressed and standardised^37^ to reach the full potential of such an innovative and exciting technology. Whereas cybersecurity measures are of the utmost consideration, the chances of hacking while performing surgery-remote interface with DTAS or manipulating patient data are unknown. Consequently, these will raise concerns for the most robust security protocols and real-time monitoring systems to avoid such cyberattacks during surgery^84^.

### Surgeon acceptance

One of the substantial obstacles to overcome in implementing DTAS is acceptance by surgeons. The medical field usually has some resistance to new technologies, which one could witness with the introduction of robotic-assisted surgeries and computer-assisted systems^86^. Many times, surgeons are more comfortable with traditional methods and may show a little reluctance to be on systems that they perceive as complex, time-consuming, or hard to master. This is particularly true in high-stakes surgeries, where trust in technology can be difficult to establish^87^. For DTAS to gain broader acceptance, surgeon training and peer advocacy will be crucial. Demonstrating the complementary role of DTAS in improving patient outcomes and enhancing surgical precision rather than replacing the surgeon’s skills will help overcome these barriers. Gradual exposure to the technology, hands-on training, peer-led demonstrations, and patient-centred co-creation will increase surgeon confidence and help enhance overall acceptance of DT-based systems^88,89^.

## Conclusions

Overall, DT technology in surgery has the potential to: (1) assist the surgical team in all stages of surgery for enhanced precision, safety, and patient care, (2) democratise surgery through improved telesurgery, and (3) provide advanced surgical training practices to novice and experienced practitioners alike. Despite these benefits, due to the infancy of DT in this sector, several technical, ethical, and regulatory challenges remain to be addressed prior to its successful clinical translation. Nonetheless, we envisage that in the forthcoming decade through collaborative inter- and multi- disciplinary academic efforts and driven by the substantial investment by major industrial players in robotic as well as DT and AI technologies, DTAS will become instrumental as we progress further into the era of digital surgery.
